# Supplementary material for: Measurement of Anti-TNF Biologics in Serum Samples of Pediatric Patients: Comparison of Enzyme-Linked Immunosorbent Assay (ELISA) with a Rapid and Automated Fluorescence-Based Lateral Flow Immunoassay
Source: Pharmaceutics. 2025 Mar 26;17(4):421. doi: 10.3390/pharmaceutics17040421 (PMC12030656; doi:10.3390/pharmaceutics17040421)
Supplement: Supplementary file 1 [file pharmaceutics-17-00421-s001.zip › Supplementary Figure S1.docx]

**Supplementary Figure S1.** Analysis of residuals for the linear regression of (**A**) infliximab (IFX) and (**B**) adalimumab (ADL) drug levels measured with both AFIAS and ELISA methods.

**A)**

**B)**
