# Supplementary material for: Measurement of Anti-TNF Biologics in Serum Samples of Pediatric Patients: Comparison of Enzyme-Linked Immunosorbent Assay (ELISA) with a Rapid and Automated Fluorescence-Based Lateral Flow Immunoassay
Source: Pharmaceutics. 2025 Mar 26;17(4):421. doi: 10.3390/pharmaceutics17040421 (PMC12030656; doi:10.3390/pharmaceutics17040421)
Supplement: Supplementary file 1 [file pharmaceutics-17-00421-s001.zip › Supplementary Table S3.docx]

**Supplementary Table S3.** Paired concentrations of infliximab (IFX) and adalimumab (ADL) drug levels measured with both AFIAS and ELISA assay.
