# Supplementary material for: Measurement of Anti-TNF Biologics in Serum Samples of Pediatric Patients: Comparison of Enzyme-Linked Immunosorbent Assay (ELISA) with a Rapid and Automated Fluorescence-Based Lateral Flow Immunoassay
Source: Pharmaceutics. 2025 Mar 26;17(4):421. doi: 10.3390/pharmaceutics17040421 (PMC12030656; doi:10.3390/pharmaceutics17040421)
Supplement: Supplementary file 1 [file pharmaceutics-17-00421-s001.zip › Supplementary Tables S1-2.docx]

**Supplementary Table S1**. Analytical specifications of AFIAS and ELISA kits used for quantification of Infliximab and Adalimumab in serum samples.

| **Specification** | **AFIAS Infliximab** | ***IDK*monitor^®^ Infliximab drug levels** | **AFIAS Adalimumab** | ***IDK*monitor^®^ Adalimumab drug levels** |
| --- | --- | --- | --- | --- |
| Type of Assay | Fluorescence immunoassay (FIA) | ELISA | Fluorescence immunoassay (FIA) | ELISA |
| Type of Determination | Quantitative | Quantitative | Quantitative | Quantitative |
| Limit of Blank (LoB) | 0.03 µg/mL | 0.002 µg/mL | 0.06 µg/mL | 0.002 µg/mL |
| Limit of Detection (LoD) | 0.12 µg/mL | 0.003 µg/mL | 0.09 µg/mL | 0.003 µg/mL |
| Lower Limit of Quantitation (LLoQ) | 0.20 µg/mL | 0.003 µg/mL | 0.20 µg/mL | 0.003 µg/mL |
| Measurement range | 0.20-50 µg/mL | 0.40-45.0 µg/mL | 0.20-50 µg/mL | 0.45-45.0 µg/mL |
| Sample type | Whole blood, plasma and serum | Plasma and serum | Whole blood, plasma and serum | Plasma and serum |
| Anticoagulant | EDTA, Sodium citrate, lithium heparin, sodium heparin | EDTA | EDTA, Sodium citrate, lithium heparin, sodium heparin | EDTA |
| Time to results | 10 minutes | 3 hours | 10 minutes | 3 hours |
| **Storage and Stability**  Component  Storage Temperature  Storage Duration (condition) | Cartridge  2-30 °C  20 months (unopened)  1 month (released) | Plate and reagents  2-8 °C  Until expiration (unopened and/or released) | Cartridge  2-30 °C  20 months (unopened)  1 month (released) | Plate and reagents  2-8 °C  Until expiration (unopened and/or released) |

**Supplementary Table S2**. Analytical specifications of AFIAS and ELISA kits used for detection of Total antibodies against Infliximab in serum samples.

| **Specification** | **AFIAS Total Anti-Infliximab** | ***IDK*monitor^®^ Infliximab total ADA** |
| --- | --- | --- |
| Type of Assay | Fluorescence immunoassay (FIA) | ELISA |
| Type of Determination | Semi-quantitative | Semi-quantitative |
| Limit of Blank (LoB) | 1.25 AU/mL | 3.12 AU/mL |
| Limit of Detection (LoD) | 7.99 AU/mL | 6.93 AU/mL |
| Working range | 8-250 AU/mL | N/A |
| Negative/Positive cut-off value | 10 AU/mL | 10 AU/mL |
| Sample type | Plasma and serum | Plasma and serum |
| Anticoagulant | EDTA, Sodium citrate | EDTA |
| Time to results | 10 minutes | 3 hours |
| **Storage and Stability**  Component  Storage Temperature  Storage Duration (condition) | Cartridge  2-30 °C  20 months (unopened)  1 month (released) | Plate and reagents  2-8 °C  Until expiration (unopened and/or released) |

Abbreviations: ADA= Anti-Drug Antibody; N/A= Not Applicable
